# Binding and Sensing Properties of a Hybrid Naphthalimide–Pyrene Aza-Cyclophane towards Nucleotides in an Aqueous Solution

**DOI:** 10.3390/molecules26040980

**Published:** 2021-02-12

**Authors:** Aleksandr M. Agafontsev, Aleksandr S. Oshchepkov, Tatiana A. Shumilova, Evgeny A. Kataev

**Affiliations:** 1 N. N. Vorozhtsov Institute of Organic Chemistry SB RAS, 9th Lavrentiev Avenue, 630090 Novosibirsk, Russia; agafon@nioch.nsc.ru; 2 Institute of Chemistry, Technische Universität Chemnitz, 09107 Chemnitz, Germany; aleksandr.oshchepkov@chemie.tu-chemnitz.de (A.S.O.); sh.tat.93@gmail.com (T.A.S.); 3 Department of Chemistry and Pharmacy, University of Erlangen-Nürnberg, Nikolaus-Fiebiger-Str. 10, 91058 Erlangen, Germany

**Keywords:** cyclophane, nucleotide recognition, supramolecular recognition, host–guest chemistry, fluorescent probe

## Abstract

Selective recognition of nucleotides with synthetic receptors is an emerging direction to solve a series of nucleic acid-related challenges in biochemistry. Towards this goal, a new aza-cyclophane with two different dyes, naphthalimide and pyrene, connected through a triamine linker has been synthesized and studied for the ability to bind and detect nucleoside triphosphates in an aqueous solution. The receptor shows Foerster resonance energy transfer (FRET) in fluorescence spectra upon excitation in DMSO, which is diminished dramatically in the presence of water. According to binding studies, the receptor has a preference to bind ATP (adenosine triphosphate) and CTP (cytidine triphosphate) with a “turn-on” fluorescence response. Two separate emission bands of dyes allow one to detect nucleotides in a ratiometric manner in a broad concentration range of 10^−5^–10^−3^ M. Spectroscopic measurements and quantum chemical calculations suggest the formation of receptor–nucleotide complexes, which are stabilized by dispersion interactions between a nucleobase and dyes, while hydrogen bonding interactions of nucleobases with the amine linkers are responsible for selectivity.

## 1. Introduction

**Figure 1:**
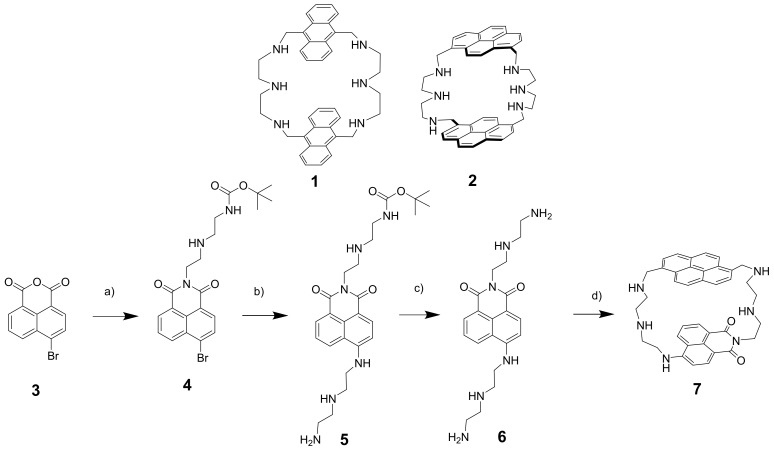
Aza-cyclophanes **1** and **2** previously studied for nucleotide binding and sensing. Synthetic route to receptor **7**: (**a**) mono-BOC-diethylenetriamine, 70 °C, reflux in EtOH (90%); (**b**) diethylenetriamine, toluene, reflux (57%); (**c**) CH_2_Cl_2_-trifluoroacetic acid, r.t. (quant.); (**d**) Pyrene-1,6-dicarbaldehyde, CH_3_OH, 50 °C; NaBH_4_ (19%).

Nucleotides are abundant in living systems and play important roles [1]. First of all, they are constituents of DNA and RNA structures and, thus, can be targeted to affect genetic information transfer. ATP is involved in cell energy conversion in almost all metabolic cycles of living organisms [2]. Biosensors such as luciferin-luciferase bioluminescence assay, green fluorescent protein-based probes and electrochemical sensors belong to the established methods of nucleotide detection [3]. However, small-molecule-based fluorescent probes represent a much cheaper solution to the problem of cellular detection of nucleotides and their quantification. It is a great challenge to design a fluorescent molecule that converts a nucleotide recognition event into a readable analytical signal [4]. During the last two decades, a series of new fluorescent probes have been developed and tested [2,5,6,7,8,9,10]. Only a few of those probes can detect nucleotides in a ratiometric manner. Such probes are based on pyrene-containing macrocycles, which are able to form excimer complexes [11], metal-based systems [12,13,14,15,16], synthetic receptors [17,18,19,20] and proteins [21]. Recently, we also reported on a series of anthracene- and pyrene-based cyclophanes which function as ratiometric probes for nucleotides (Figure 1, receptors **1** and **2**). The cyclophanes with a triamine linker demonstrated much better selectivity for ATP than those containing oxygen atoms in the linker structure. The mechanism of ratiometric response was explained in terms of a bellows-like sensing mechanism. Two pyrene rings formed an excimer in a buffered solution in a free state, while binding of ATP disrupted pyrene–pyrene interaction in the ground and excited states.

Naphthalimide derivatives are known to show Foerster resonance energy transfer (FRET) with pyrene [22]. Thus, a cyclophane that contains both naphthalimide and pyrene is highly attractive for ratiometric nucleotide detection. There are several mechanisms of fluorescent changes to be expected for such an unsymmetrical system. First, the signal ratio will be dependent on the distance between two fluorophores in solution. Second, the neighboring amine groups will quench the fluorescence of both naphthalimide and pyrene through photoinduced electron transfer (PET). As we demonstrated in our previous report, binding of a nucleotide often shifts the p*K_a_* values of interacting amines with phosphate/nucleobase, which, in turn, hinders the PET process [23].

Herein, we present the design, synthesis and binding studies of the first hybrid naphthalimide–pyrene aza-cyclophane in an aqueous solution. The receptor binds nucleotides with the preference for ATP (adenosine triphosphate) and CTP (cytidine triphosphate) in both binding and sensing experiments, and it is completely silent towards pyrophosphate.

## 2. Results and Discussion

The target receptor was synthesized successfully with only one protection step, starting from 4-bromo-naphthanhydride (**3**) and pyrene-1,6-dicarboxaldehyde. Compound **5** was deprotected to obtain **6**—a starting material for the cyclophane synthesis. Schiff base condensation followed by reduction with sodium borohydride yielded **7** at 19%.

To analyze the binding and sensing properties of receptor **7**, we started with an analysis of the receptor behavior in different solvents and in the pH region 3‒8.

**Figure 2:**
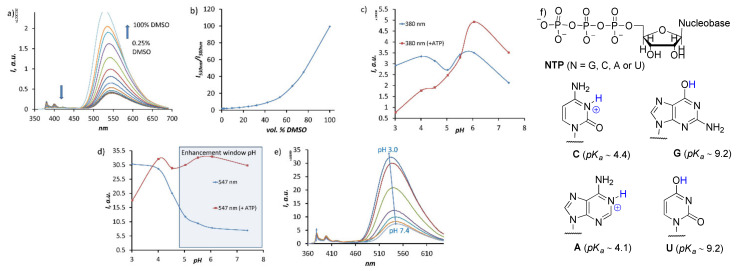
(**a**) Fluorescence changes, observed during the dilution of DMSO solution, of **7** (0.01 mM) with a 10-mM TRIS buffer. Conditions: pH 7.4, 100 mM NaCl, ex: 350 nm. (**b**) Changes in intensity ratio *I_530nm_/I_380nm_* with increasing amounts of DMSO portion. (**c,d**) Intensity–pH ratio detected at 380 and 547 nm, respectively. (**e**) Fluorescence spectra of **7** (0.01 mM) at different pH values with constant receptor concentration. (**f**) Structures of nucleoside triphosphates together with *pK_a_* values of the acidic functions are shown in blue [25].

The fluorescence spectra of the receptor in an aqueous solution and in DMSO appeared to be dramatically different. Two intensity bands at 380 and 550 nm were observed in an aqueous solution, which correspond to the emission of the pyrene and naphthalimide fragments, respectively. However, in DMSO, the emission of the pyrene fragment was much lower, likely due to the FRET process between the fluorophores. An efficient FRET process could arise when two dyes are close to each other and form strong π–π interactions. To prove this fact, we titrated the DMSO solution of **7** with water and observed an increase in the pyrene emission band and a decrease in the naphthalimide band (Figure 2a,b). These solvent-dependent changes indicate that upon increasing the water content in DMSO, the amine linkers in the receptors are protonated and thus destabilize the stacked conformation of the cyclophane. As can be seen in Figure 2b, the ratio of band intensities increases dramatically from 2 to 100 with an increasing portion of DMSO. The ^1^H-^1^H ROESY NMR experiments in DMSO-*d6* also provided proof of the through-space interactions of the pyrene and naphthalimide dyes (cf. Figure S8). Next, we investigated how the pH of the solution affects the properties of the receptor. Solutions with different pH values were prepared by mixing appropriate amounts of NaOH and CH_3_COOH aqueous solutions, keeping the acetate concentration constant (50 mM). Fluorescence measurement of the receptor solutions at different pH values revealed interesting trends, shown in Figure 2c–e. The fluorescence pH-dependence for two emission bands appeared to be different. Pyrene emission is not considerably affected by pH changes, indicating a relatively weak PET process from the amine groups to pyrene. However, the pyrene emission reaches the maximum value at pH 6.3 in the presence of ATP. Interestingly, the response of the pyrene is different at neutral (enhancement) and at acidic (quenching) pH values, which can originate from different protonation states of host and guest molecules (Figure 2f). On the contrary, naphthalimide is much more sensitive to pH changes. Its fluorescence increases upon protonation and interaction with ATP is observed in a broad pH range of 5‒8. The results of the spectroscopic measurements suggest that secondary amine groups, which are responsible for fluorescence enhancement, have p*K_a_* values around five (corresponds to the conjugated acid, Figure 2d). This value is in agreement with the 3rd or 4th protonation constant of known cyclic polyammonium receptors [24]. Overall, the pH-dependent measurements already demonstrate that the receptor **7** is suitable for nucleotide detection at neutral pH values.

**Figure 3:**
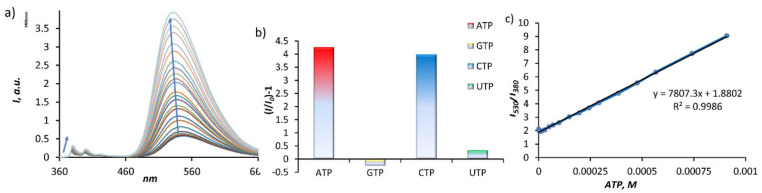
(**a**) Fluorescence changes observed during the titration of **7** (0.01 mM) with ATP (1 mM) in a 10-mM TRIS buffer (2% vol. DMSO, 100 mM NaCl, pH 7.4, ex.: 350 nm). (**b**) Fluorescence response of **7** towards nucleoside triphosphates. (**c**) Changes in intensity ratio *I_530nm_*/*I_380nm_* with increasing amounts of ATP portion.

To understand the selectivity of **7** in binding and sensing of nucleotides, we conducted fluorescent titrations with four nucleoside triphosphates: ATP, CTP, GTP, and UTP. Fitting the experimental data with the HypSpec program [26] yielded the following binding constants (log*K,* 1:1 complex): ATP, 4.80(4); CTP, 4.53(5); GTP, 3.66(3); UTP, 3.23(2). According to the fitting analysis of the titration data by HypSpec and Job Plots (UV–Vis), nucleotides are bound with a stepwise 1:1 and 1:2 stoichiometry. However, the second binding event is much weaker and, in some cases, could not be precisely determined (Figures S12–S14). The binding constants obtained from UV–Vis and fluorescence titrations were in a good agreement (Table S1). The general behavior of the receptor **7** in the presence of nucleotides features a fluorescence enhancement observed for both emission bands at 380 and 550 nm. As can be seen in Figure 3a, the fluorescence enhancement of the naphthalimide moiety is much stronger than that of the pyrene moiety and reaches six- and fivefold for ATP and CTP, respectively (Figure 3b). An expected behavior was observed for GTP, which quenched the fluorescence of both fluorophores due to the PET process. The selectivity of **7** for adenine- and cytosine-containing nucleotides can be explained by the fact that these nucleotides have the lowest p*K_a_* values and, thus, can form hydrogen bonds with the amine linkers. Complexation-induced formation of hydrogen bonds between a nucleobase and an amine linker can also serve as an argument to explain a strong fluorescence enhancement.

The fluorescence titrations revealed that upon binding, the emission bands undergo shifting with increasing amounts of nucleotides. The naphthalimide band undergoes a hypsochromic shift in the presence of all nucleotides. Remarkably, such a shift was already observed upon the receptor protonation (Figure 2e). This fact provides evidence that binding of the negatively charged species (nucleoside triphosphate) leads to protonation of the secondary amine groups adjacent to the naphthalimide ring. We also observed such a hypsochromic shift recently in our naphthalimide-based receptors for anions [27].

The bathochromic shift of the pyrene emission band is stronger for ATP than that for the other studied nucleotides, which were smaller or absent. Interestingly, the protonation of **7** does not induce any shift in pyrene emission. Thus, we assign the pyrene bathochromic shift to the adenine–pyrene π–π interactions, which are likely to be strong in the buffered aqueous solution. We have already observed similar spectroscopic changes for a pyrene-based ATP-selective receptor [11]. It is likely that this interaction also contributes to the observed selectivity of **7** for ATP over other nucleotides. We attempted to carry out ^1^H-NMR titration of **7** with ATP, but the low solubility of the complex hampered these studies. The addition of only one equivalent of ATP to **7** (5 × 10^−4^ M solution in a mixture of 25% DMSO and 10 mM TRIS buffer, 100 mM NaCl, pH 7.4) resulted in precipitation of the complex. The titration was possible only with AMP (adenosine monophosphate)—a guest with lower charge and, thus, weaker affinity. The addition of up to 10 equiv of AMP induced a downfield shift of pyrene and naphthalimide signals, while the signals of adenine underwent upfield shifts (Figure S11). Thus, this experiment additionally supports the inclusion of adenine between the two dyes.

The results show that FRET is minimal in an aqueous solution, since the protonated amine linkers connected to pyrene and naphthalimide repel and destabilize stacking interactions. This is an opposite situation to that observed by us for the recently reported bis-pyrene macrocycle, for which the pyrene excimer (stacked form) was detected in a broad pH range, despite the presence of protonated linkers. A comparison of the symmetric pyrene–pyrene cyclophane and unsymmetrical pyrene–naphthalimide receptors suggests that the excimer pyrene-pyrene complex in water is much stronger than the donor–acceptor pyrene-naphthalimide complex. Nevertheless, the new receptor still allows the detection of nucleotides in a ratiometric manner. As can be inferred from Figure 3c, the band ratio *I*_550nm_/*I*_380nm_ increases linearly in a broad concentration range between 10^−5^ and 10^−3^ M, which can be used for the assessment of ATP or CTP concentrations. The calculated limit of detection is 0.08 mM (see Supplementary Materials).

**Figure 4:**
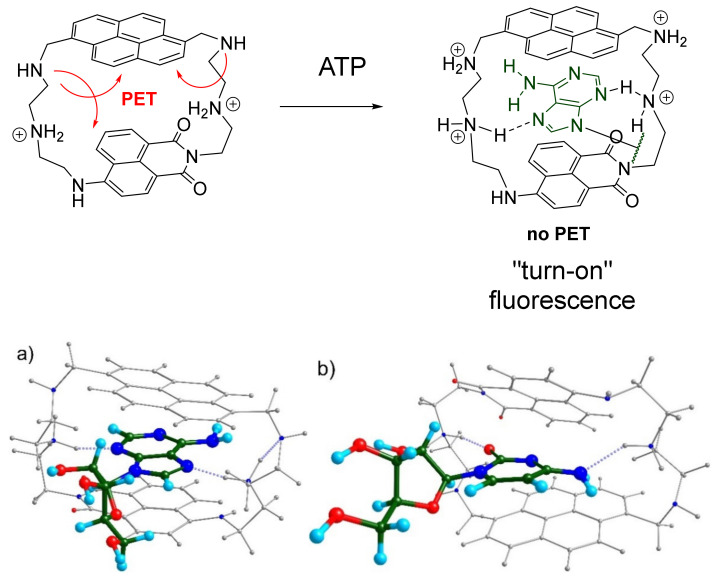
Proposed mechanism of the “turn-on” fluorescent response of **7** towards ATP. Optimized molecular structures of **7** with (**a**) adenosine and (**b**) cytidine, showing hydrogen bonding interactions of nucleobases with amine linkers.

Based on our spectroscopic measurements, we proposed the sensing mechanism of ATP with **7** in a buffered aqueous solution, which is shown in Figure 4. In a free state, the receptor is partially protonated, likely in the adjacent position to the imide fragment. The p*K_a_* value of this secondary amine group is lower than that of other linkers, as it was found in our previous work [28]. According to the literature data, **7** should be at least twofold or threefold protonated at pH 7.4. We have listed, in Figure 2f, the p*K_a_* values of four nucleobases and their conjugated acids, which fit into the protonation and sensing windows of the cyclophane (pH 4–9). Adenine and cytosine nucleobases are known to have low p*K_a_* values in comparison with guanine and cytosine and they can even be shifted towards basic values because we use negatively charged nucleoside triphosphates [25]. Thus, it is suggested that the coordination of ATP and CTP inside the aza-cyclophane leads to a higher degree of cyclophane protonation as compared to the binding with GTP and UTP. Therefore, this protonation process partially blocks possible PET processes from amine groups to the dyes, which subsequently leads to a fluorescence enhancement.

To understand the coordination mode of adenine and cytosine with the receptor, we performed quantum chemical calculations of the complexes with adenosine and cytidine, taken as model compounds for ATP and CTP, respectively. The molecular geometries of the 2x-protonated receptor were optimized by a parametrized model involving dispersion interactions [29,30], starting from a limited set of conformations from molecular dynamics. The calculations were performed using the software “Priroda” developed by D. Laikov [31,32]. As can be seen in Figure 4a,b, the nucleobases are coordinated inside the cyclophane and the host–guest complex is stabilized by dispersion and hydrogen bonding interactions. Interestingly, the hydrogen bonds formed by amine linkers are different in the cases of adenine and cytosine nucleobases. Cytosine is stabilized by hydrogen bonds formed with the oxygen atom and the NH_2_ residue. However, in the case of adenine, the NH_2_ group is not involved in the bonding because of lower basicity as compared to other nitrogen atoms possessing stronger basicity. This fact may explain the slight preference of the receptor for adenine over cytosine.

## 3. Materials and Methods

All the solvents were dried according to standard procedures. Reactions were performed in oven-dried round bottom flask. Crude products were purified by column chromatography on silica gel 100–200 mesh. TLC plates were visualized by exposure to ultraviolet light and/or by exposure to acidic ethanolic solution of ninhydrin followed by heating (<1 min) on a heat gun (~250 °C). Organic solutions were concentrated on rotary evaporator at 35–40 °C. NMR Spectra were measured on ASCEND 600 FT (Chemnitz, Germany) spectrometer (Bruker Corp., Billerica, MA, USA), 600 MHz for ^1^H-NMR and 150.9 MHz for ^13^C-NMR. The chemical shifts are reported in δ (ppm) relative to external standards (solvent residual peak). The solvent used is reported for each spectrum. Mass Spectra: Finnigan MAT TSQ 7000 (ESI). Absorption spectra were measured in 1 cm quartz cuvettes with Varian Cary BIO 50 UV/VIS/NIR Spectrometer. Emission spectra were recorded with aqueous buffered solution in 1 cm quartz cuvettes (Hellma, Müllheim, Germany) on a FluoroMax 4 (Horiba, CA, USA) with a temperature control. pH-Measurements were carried out on a Mettler Toledo G20 Titrator equipped with a DG115-SC pH-electrode. The electrode was calibrated with standard calibrating solutions from Mettler Toledo. The reaction vessels were kept at constant temperature 23 °C. The starting compounds were purchased from TCI (Eschborn, Germany), Sigma-Aldrich (Munich, Germany) and Acros Chemicals (Geel, Belgium).

First, 1,6-dibromopyrene and 1,8-dibromopyrene were synthesized according to methods in the literature, without modifications [33]. We used previously published procedures to synthesize pyrene-1,8-dicarbaldehyde or pyrene-1,6-dicarbaldehyde [34] and tert-butyl (2-((2-aminoethyl)amino)ethyl)carbamate [35].

Dialdehyde was synthesized using the modified procedure. A solution of *n*-butyllithium in hexane (2.5 M, 6 equiv) was added dropwise to the solution of the 1,6- and 1,8-dibromopyrene mixture (1:1 molar ratio obtained from the previous reaction) in anhydrous THF (tetrahydrofuran) under an argon atmosphere at −50 °C. The reaction was stirred for 20 min at this temperature and then warmed up to 25 °C and stirred for an additional 1 h. The reaction mixture was cooled to −40 °C and DMF (dimethylformamide, 6 equiv) was added dropwise to the solution. The mixture was stirred at room temperature for 12 h before water was added. After that, THF was removed under reduced pressure. The resulting residue was suspended in 100 mL H_2_O and 200 mL DCM (dichloromethane) and the aqueous phase was extracted three times with 200 mL CH_2_Cl_2_. The organic phases were combined and dried over Na_2_SO_4_. The solvents were removed under reduced pressure. The residue was purified by gradient column chromatography on silica gel, starting with a DCM/hexane 1:1 mixture as the eluent and decreasing the hexane portion until pure DCM was used. The first fraction contained pyrene-1,6-dicarbaldehyde (25% yield), and the second fraction contained pyrene-1,8-dicarbaldehyde (21% yield).

### 3.1. Tert-Butyl-(2-((2-(6-bromo-1,3-dioxo-1H-benzo[de]isoquinolin-2(3H)-yl)ethyl)amino)ethyl)carbamate (**4**)

Briefly, 4-Bromo-1,8-naphthalic anhydride (**3**, 5.17 g, 18.66 mmol) and *tert*-butyl (2-((2-aminoethyl)amino)ethyl)carbamate (3.79 g, 18.66 mmol) were mixed together in a 250-mL round-bottom flask equipped with a reflux condenser and a stirring bar. To the reaction mixture, 100 mL ethanol was added and the resulting mixture was stirred at 70 °C for 5 h. After cooling down to room temperature, THF was removed under reduced pressure. The resulting residue was suspended in 150 mL H_2_O and 150 mL CHCl_3_, and the aqueous phase was extracted twice with 150 mL CHCl_3_. The organic phases were combined and dried over Na_2_SO_4_. The solvents were removed under reduced pressure. The residue was purified by column chromatography on silica gel using a gradient: CHCl_3_:EtOH = 10:1 to CHCl_3_:EtOH = 4:1.The product was obtained as a white powder. Yield: 90%; ^1^H-NMR (600 MHz, CDCl_3_) δ 8.60 (d, 1H, *J* = 7.2 Hz), 8.52 (d, 1H, *J* = 8.3 Hz), 8.36 (d, 1H, *J* = 7.6 Hz), 7.98 (d, 1H, *J* = 8.0 Hz), 7.79 (dd, 1H, *J* = 8.0; 7.6 Hz), 4.30 (dd, 2H, *J* = 6.5; 6.5 Hz), 3.20 (m, 2H), 3.02 (m. 2H), 2.83 (m, 2H), 1.37 (s, 9H); ^13^C-NMR (CDCl_3_, 25 °C, δ, ppm.): 27.3, 37.1, 37.2, 51.1, 52.3, 77.8, 121.9, 122.8, 127.2, 126.0, 131.6, 132.2, 1321.6, 133.4, 134.5, 134.6, 156.8, 164.8, 164.9. HRMS (ESI-TOF) *m*/*z*: calcd. for C_21_H_25_BrN_3_O_4_ [M + H]^+^ 462.0950; found: *m*/*z* = 462.0935.

### 3.2. Tert-Butyl-(2-((2-(6-((2-((2-aminoethyl)amino)ethyl)amino)-1,3-dioxo-1H-benzo[de]isoquinolin-2(3H)-yl)ethyl)amino)ethyl)carbamate (**5**)

Tert-butyl-(2-((2-(6-bromo-1,3-dioxo-1H-benzo[de]isoquinolin-2(3H)-yl)ethyl)amino)ethyl)carbamate (**4**, 2.22 g, 4.80 mmol) and ethylenediamine (9.00 g, 87.0 mmol) were mixed together in a 50-mL round-bottom flask equipped with a reflux condenser and a stir bar. To this reaction mixture, 5 mL toluene was added and the resulting mixture was stirred at room temperature; the reaction progress was monitored by TLC (Thin-layer chromatography). The solvents were removed under reduced pressure. The residue was purified by gradient column chromatography on silica gel using the following eluents: CHCl_3_:EtOH:NH_3_ aq. sol. = 100:100:0 to CHCl_3_:EtOH: NH_3_ aq. sol. = 100:100:10. The product obtained was an orange powder. Yield: 57%; ^1^H-NMR (600 MHz, CDCl_3_) δ 8.55 (d, 1H, *J* = 7.3 Hz), 8.42 (d, 1H, *J* = 8.7 Hz), 8.17 (d, 1H, *J* = 8.7 Hz), 7.58 (dd, 1H, *J* = 7.7; 7.7 Hz), 7.65 (d, 1H, *J* = 8.7 Hz), 6.35 (t, 1H(Ar-NH), *J* = 4.3; 4.3 Hz), 5.15 (sb, 1H(NHCO)), 4.28 (dd, 2H, *J* = 5.9; 5.9 Hz), 3.42 (m, 2H), 3.18 (m, 2H), 3.08 (dd, 2H, *J* = 6.2; 6.2 Hz), 2.97 (dd, 2H, *J* = 6.2; 6.2 Hz), 2.88 (dd, 2H, *J* = 6.2; 6.2 Hz), 2.81 (dd, 2H, *J* = 6.2; 6.2 Hz), 2.75 (dd, 2H, *J* = 6.2; 6.2 Hz), 1.40 (s, 9H); ^13^C-NMR (DMSO-*d6*, 25 °C, δ, ppm.): 27.4, 36.8, 37.3, 38.6, 40.3, 48.4, 51.0, 51.3, 52.2, 77.6, 103.3, 108.1, 111.3, 121.6, 122.4, 128.7, 131.0, 130.4, 134.0, 151.4, 157.2, 165.3, 166.0. HRMS (ESI-TOF) *m*/*z*: calcd. for C_25_H_37_N_6_O_4_ [M + H]^+^ 485.2798; found: *m*/*z* = 485.2776.

### 3.3. 12,13-dihydro-11H-4,7,11,14,17-pentaaza-1(2,6)-benzo[de]isoquinolina-9(1,6)-pyrenacycloheptadecaphane-11,13-dione (**7**)

*Tert*-Butyl-(2-((2-(6-((2-((2-aminoethyl)amino)ethyl)amino)-1,3-dioxo-1H-benzo[de]isoquinolin-2(3H)-yl)ethyl)amino)ethyl)carbamate (**5**, 453 mg, 0.90 mmol) and DCM (10 mL) were mixed together in a 50-mL round-bottom flask equipped with a stir bar. To this reaction mixture, 15 mL trifluoroacetic acid was added and stirred at room temperature for 48 h. The reaction progress was monitored by TLC. The solvents were removed under reduced pressure. The residue was dissolved in 20 mL methanol. Product **6** was used without further purification.

Pyrene-1,6-dicarbaldehyde (0.90 mM, 232 mg) was placed into a round-bottom flask, and then, 400 mL of acetonitrile and 20 mL of methanol were added. The flask was heated in an oil bath at 50 °C with stirring until the dialdehyde was completely dissolved. Next, potassium carbonate (5.40 mM, 746 mg) was added. The resulting solution of deprotected amine **6** in 20 mL methanol was slowly added from a dropping funnel to the pyrene-1,6-dicarbaldehyde solution while stirring and heating at 50 °C. The reaction was completed after heating at 50 °C for 72 h. The solvent was removed under reduced pressure without heating. Methanol (250 mL) and sodium borohydride (1 g, 26 mmol) were added to the residue. The flask was heated in an oil bath at 50 °C for 3 h; then, the reaction was maintained overnight at room temperature. Methanol was removed under reduced pressure. The resulting solid was suspended in 200 mL H_2_O and 100 mL of a chloroform:ethanol = 100:10 mixture, and the aqueous phase was extracted three times with 100 mL of chloroform:ethanol 100:10 mixture. The organic phases were collected and dried with Na_2_SO_4_. The solvents were removed under reduced pressure. The residue was purified by gradient column chromatography on silica gel using the following eluents: EtOH:CHCl_3_: NH_3_ aq. sol. = 1:1:0 to ethanol:chloroform: NH_3_ aq. sol. = 100:100:4 to give an orange powder. Yield: 19%; ^1^H-NMR (600 MHz, DMSO-d_6_) δ 8.43 (d, 1H, *J* = 9.0 Hz), 8.27 (d, 1H, *J* = 7.0 Hz), 8.22 (d, 1H, *J* = 8.3 Hz), 8.07 (d, 1H, *J* = 9.5 Hz), 8.03 (d, 1H, *J* = 9.5 Hz), 7.99 (m, 2H), 7.95 (d, 1H, *J* = 8.3 Hz), 7.91 (d, 1H, *J* = 9 Hz), 7.85 (d, 1H, *J* = 7.5 Hz), 7.44 (dd, 1H, *J* = 8.7; 8.7 Hz), 7.42 (d, 1H, *J* = 8.7 Hz), 7.08 (d, 1NH, *J* = 4.5 Hz), 5.65 (d, 1H, *J* = 8.7 Hz), 4.39 (s, 2H), 4.12 (s, 2H), 4.09 (dd, 2H, *J* = 6.0; 6.9 Hz), 2.83 (m, 2H), 2.81 (m, 2H), 2.72 (m, 4H), 2.68 (m, 4H, *J* = 6.0; 6.0 Hz), 2.51 (m, 2H), 2.31 (dd, 2H, *J* = 6.2; 6.2 Hz); ^13^C-NMR (150 MHz, CD_3_OH-CDCl_3_) δ 165.2, 164.9, 149.75, 134.5, 133.4, 132.70, 130.5, 130.4, 129.7, 129.2, 129.1, 128.3, 127.7, 127.6, 127.1, 126.8, 125.5, 120.0, 124.7, 124.5, 123.5, 122.6, 122.3, 121.3, 119.5, 107.8, 103.0, 51.6, 51.0, 48.1, 47.9, 46.8, 46.7, 46.3, 46.2, 42.31, 38.7 HRMS (ESI-TOF) *m*/*z*: calcd. for C_38_H_38_N_6_O_2_ [M + H]^+^ 611.3129; found: *m*/*z* = 611.3115.

## 4. Conclusions

In conclusion, we have designed and synthesized the new unsymmetrical cyclophane **7** containing two different fluorescent dyes, pyrene and naphthalimide. The receptor was explored for the detection of nucleotides in an aqueous buffered solution. We have analyzed the conformations of the receptor in organic and aqueous solutions at different pH values. It was found that the receptor in polar aprotic solvent is in the stacked conformation (π–π interactions between pyrene and naphthalimide) and the FRET process is observed. Protonation of the amine groups in an aqueous solution, on the other hand, leads to the repulsion of dyes and the non-stacked conformation prevails. This is the opposite behavior of the receptor to that observed for symmetrical pyrene- or anthracene-based cyclophanes, for which the excimer complex is stable in an aqueous solution but can be destroyed by the addition of a nucleotide. The binding studies revealed the preference of **7** to bind ATP and CTP, which is accomplished with six- and fivefold fluorescence enhancement upon saturation with nucleotides, respectively. The receptor can be used to detect these nucleotides in a ratiometric manner in a broad range of concentrations 10^−5^–10^−3^ M.

## Supplementary Materials

The following are available online, Figure S1–S14: NMR, UV–Vis and fluorescence spectra of compounds, Table S1: measured binding constants, Table S2: Cartesian coordinates of optimized structures.

## Sample Availability

Samples of the compound **7** are available from the authors.
