# Supplementary material for: Binding and Sensing Properties of a Hybrid Naphthalimide–Pyrene Aza-Cyclophane towards Nucleotides in an Aqueous Solution
Source: Molecules. 2021 Feb 12;26(4):980. doi: 10.3390/molecules26040980 (PMC7918853; doi:10.3390/molecules26040980)
Supplement: Supplementary file 1 [file molecules-26-00980-s001.pdf]

Supporting Information

Binding and Sensing Properties of a Hybrid Naph-thalimide–Pyrene Aza-Cyclophane towards Nucleotides in an Aqueous Solution

Aleksandr M. Agafontsev,[a] Aleksandr S. Oshchepkov,[b] Tatiana A. Shumilova,[b] Evgeny A. Kataev[c]*

[a] *N. N. Vorozhtsov Institute of Organic Chemistry SB RAS, 9th Lavrentiev Avenue, 630090, Novosibirsk (Russian Federation); agafon@nioch.nsc.ru*
[b] *Institute of Chemistry, Technische Universität Chemnitz, 09107 Chemnitz (Germany); alexandr.oshch@mail.ru*
[c] *Department of Chemistry and Pharmacy, University of Erlangen-Nürnberg, Nikolaus-Fiebiger-Str. 10, 91058 Erlangen Germany*
**Correspondence: evgeny.kataev@fau.de*

Content

General 2

NMR spectra 2

^{1}H NMR titrations 8

Fluorescence and UV-Vis titrations 8

  The limit of detection (LOD) 10

  Binding constants 12

Competition experiment 12

Quantum chemical calculations 13

References 16

General

All solvents were dried according to standard procedures. Reactions were performed in oven-dried round bottom flask. Crude products were purified by column chromatography on silica gel 100-200 mesh. TLC plates were visualized by exposure to ultraviolet light and/or by exposure to acidic ethanolic solution of ninhydrin followed by heating (<1 min) on a heat gun (~250 °C). **NMR Spectra** were measured on ASCEND 600 FT spectrometer (Bruker Corp.), 600 MHz for ^{1}H NMR and 150.9 MHz for ^{13}C NMR. The chemical shifts are reported in δ [ppm] relative to external standards (solvent residual peak). The solvent used is reported for each spectrum. **Mass Spectra:** Finnigan MAT TSQ 7000 (ESI). **Absorption spectra** were measured in 1 cm quartz cuvettes with Varian Cary BIO 50 UV/VIS/NIR Spectrometer. **Emission spectra** were recorded in 1 cm quartz cuvettes (Hellma) on a FluoroMax 4 (Horiba) with a temperature control. **pH-Measurements** were carried out on a Mettler Toledo G20 Titrator equipped with a DG115-SC pH-electrode. The electrode was calibrated with standard calibrating solutions from Mettler Toledo. The reaction vessels were kept at constant temperature 23°C. The starting compounds were purchased from TCI, Sigma-Aldrich and Fisher Scientific.

NMR spectra

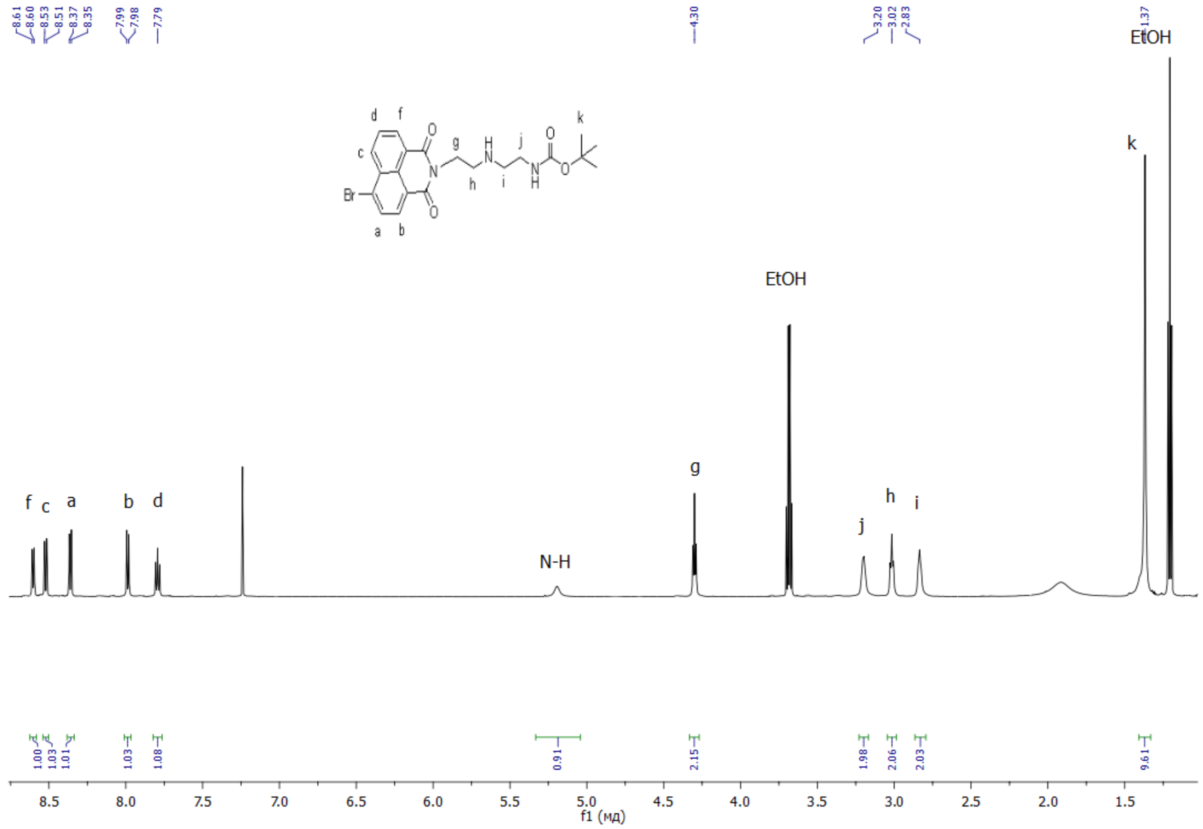

Figure S1. ^{1}H NMR spectrum (600MHz, $CDCl_3$, 298 K) of **4**.

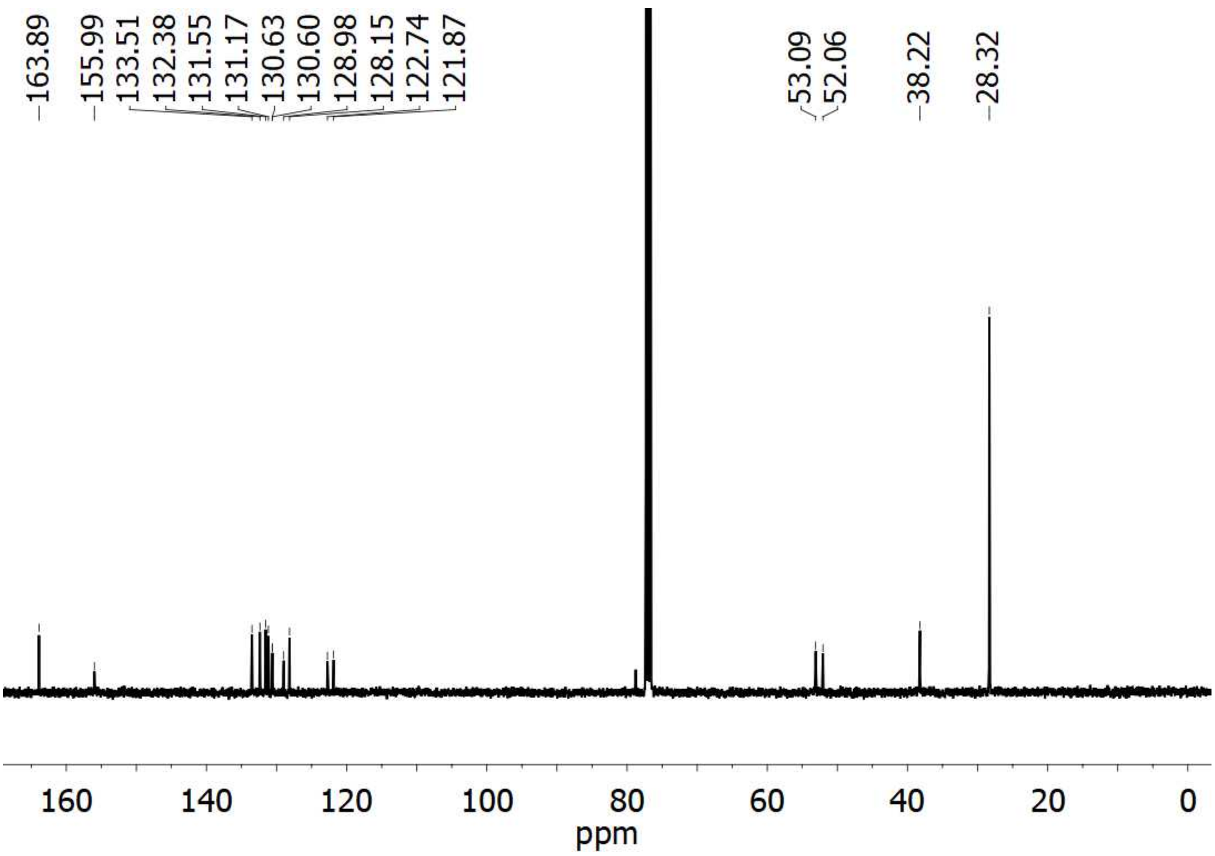

Figure S2. ^{13}C NMR spectrum (151 MHz, $CDCl_3$, 298 K) of **4**.

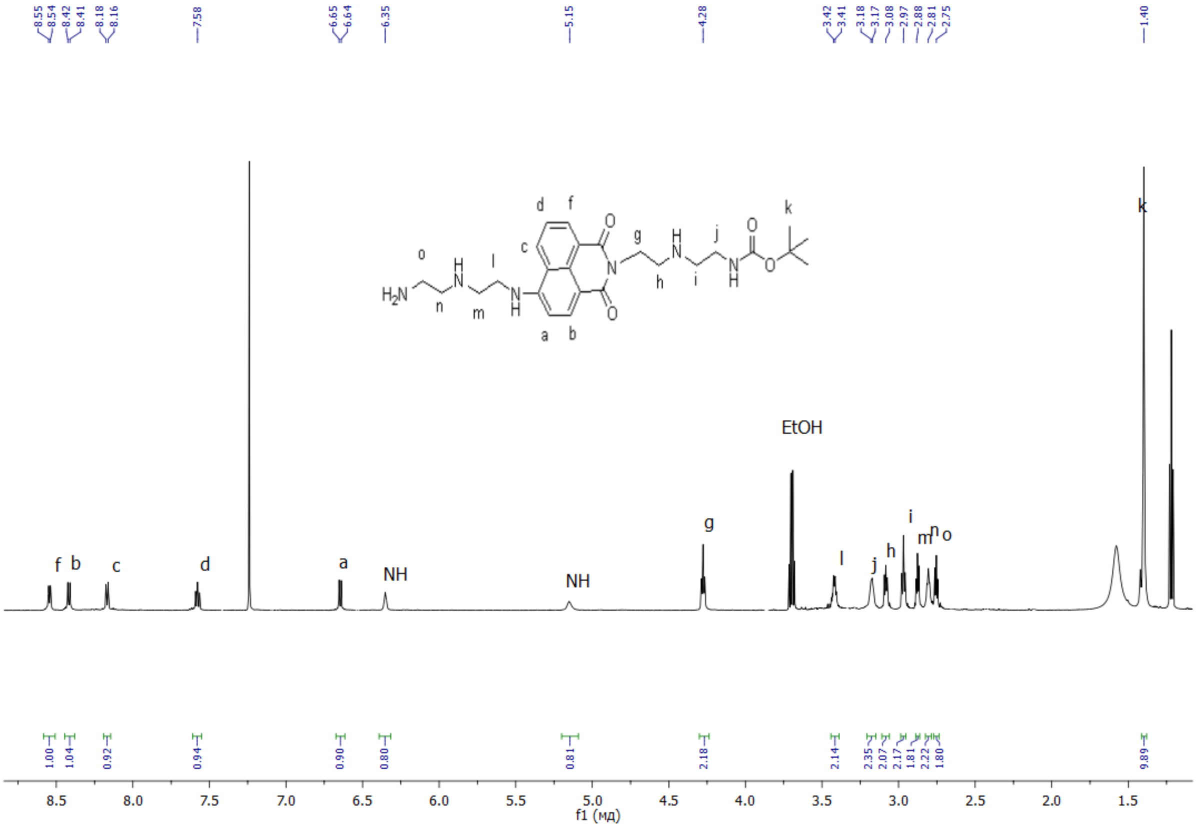

Figure S3. ^{1}H NMR spectrum (600MHz, $CDCl_3$, 298 K) of **5**.

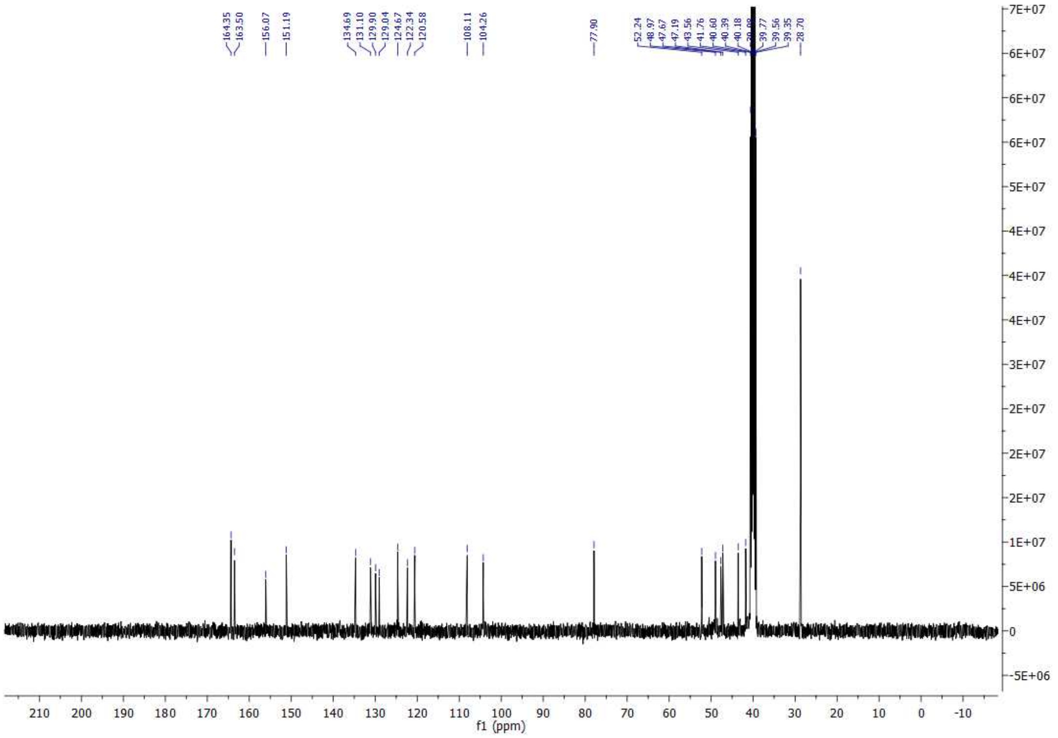

Figure S4. ^{13}C NMR spectrum (151 MHz, DMSO-*d6*, 298 K) of **5**.

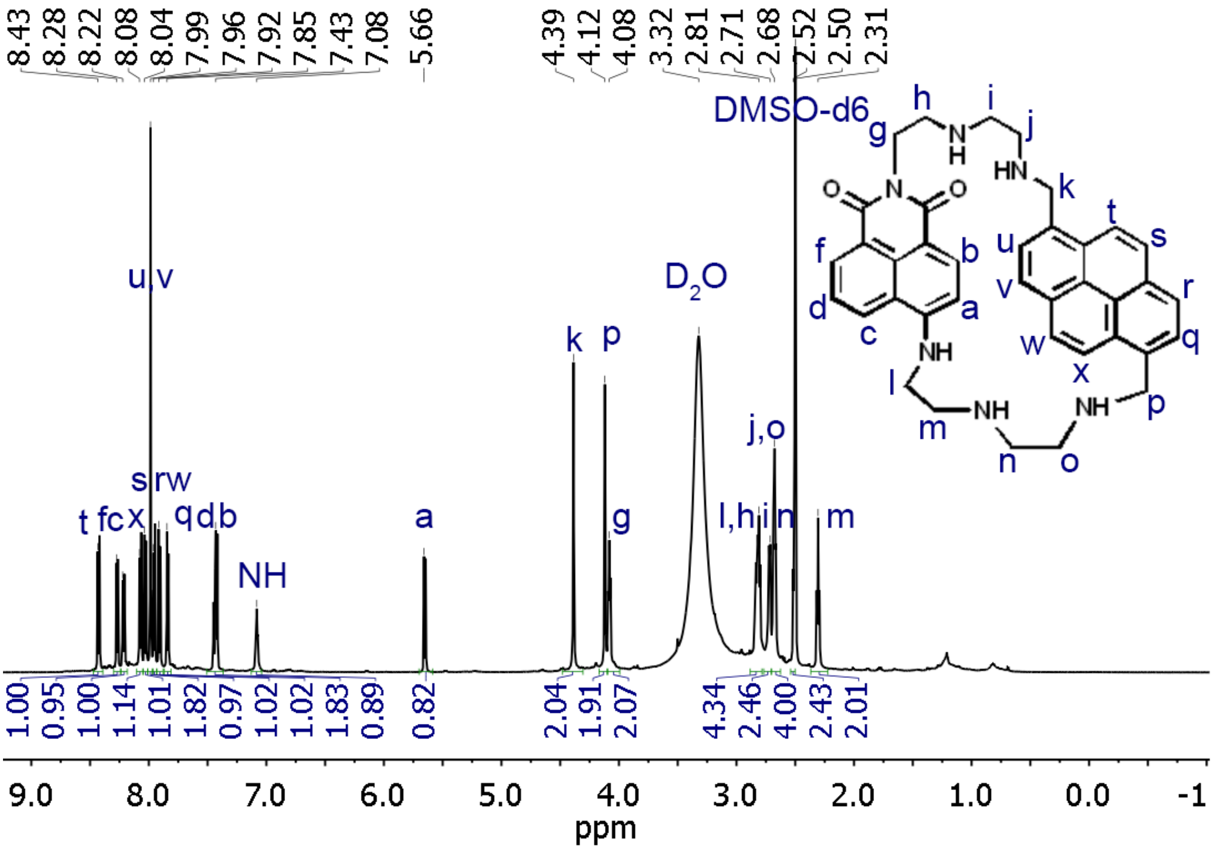

Figure S5. ^{1}H NMR spectrum (600MHz, DMSO-*d6*, 298 K) of **7**.

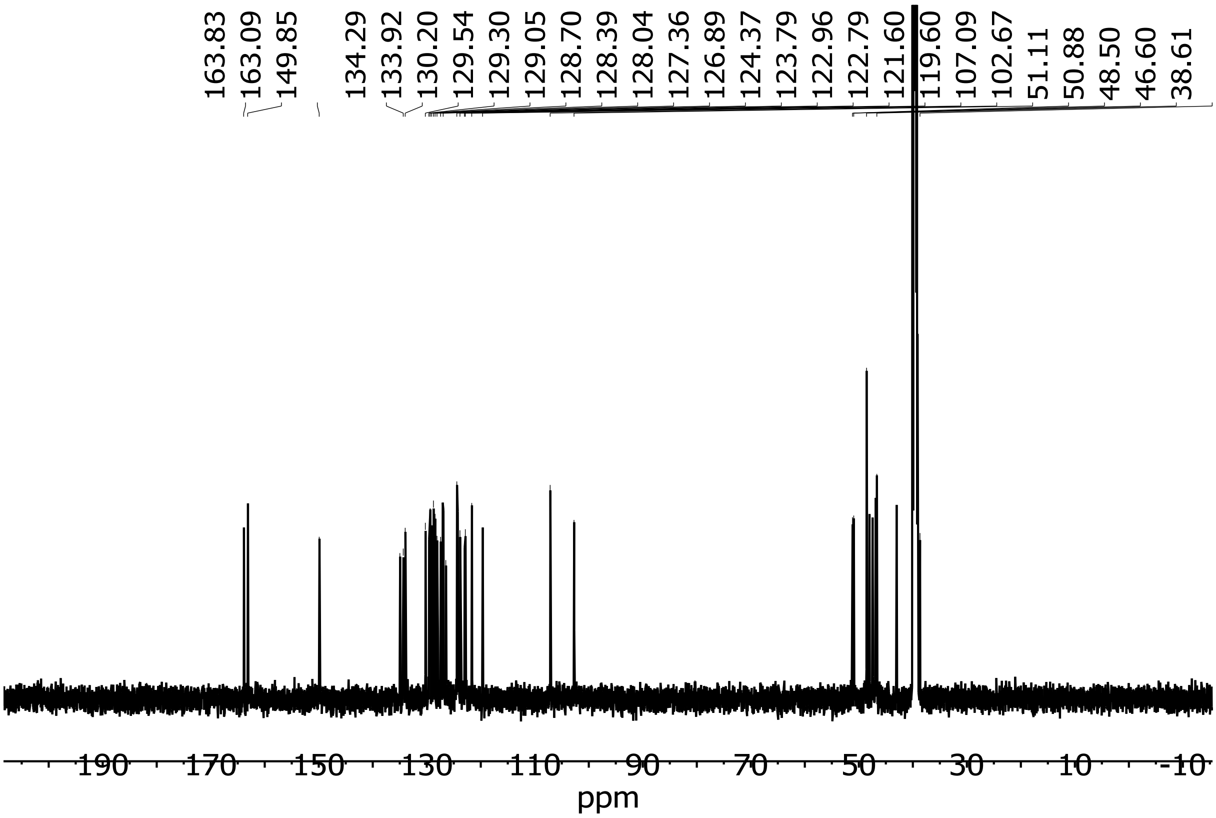

Figure S6. ^{13}C NMR spectrum (151 MHz, DMSO-*d6*, 298 K) of **7**.

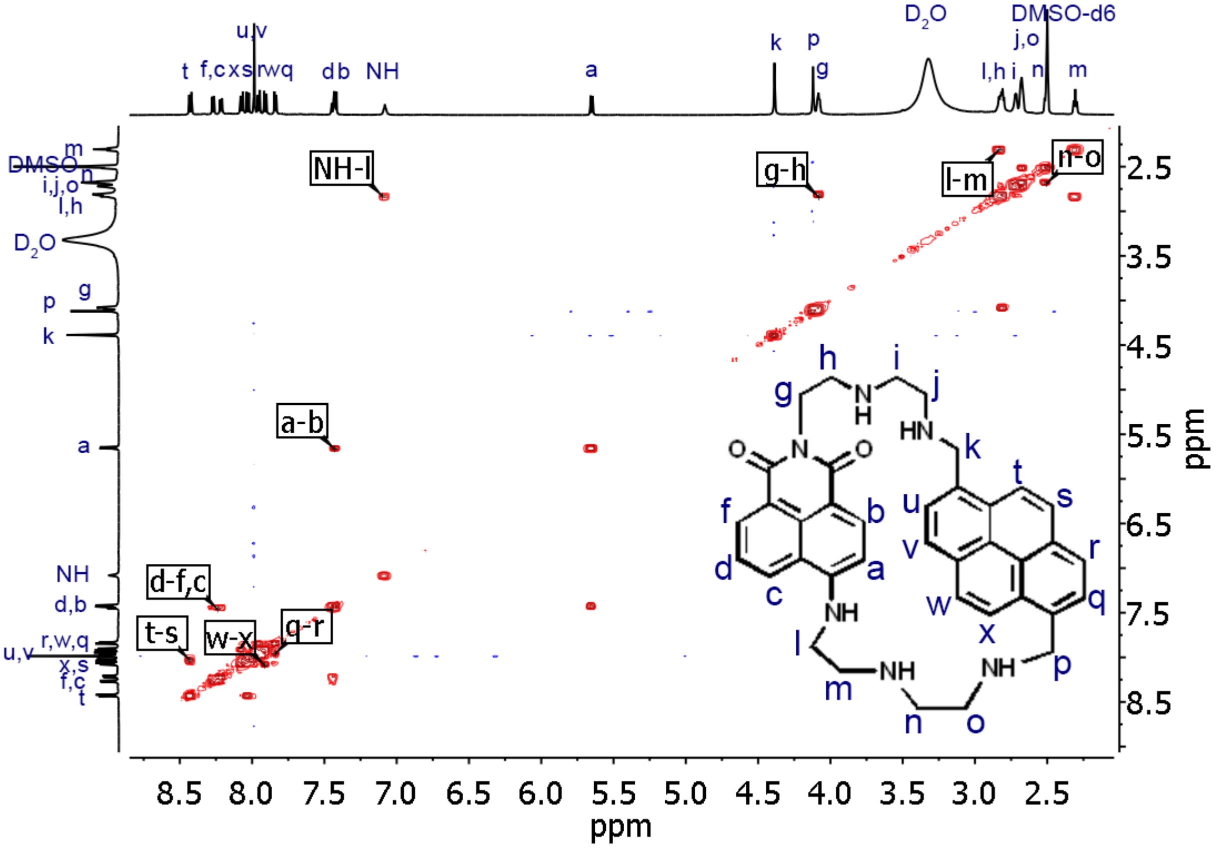

Figure S7. Annotated 2D ^{1}H-^{1}H COSY NMR spectrum (600 MHz, DMSO-*d6*, 298K) of **7**.

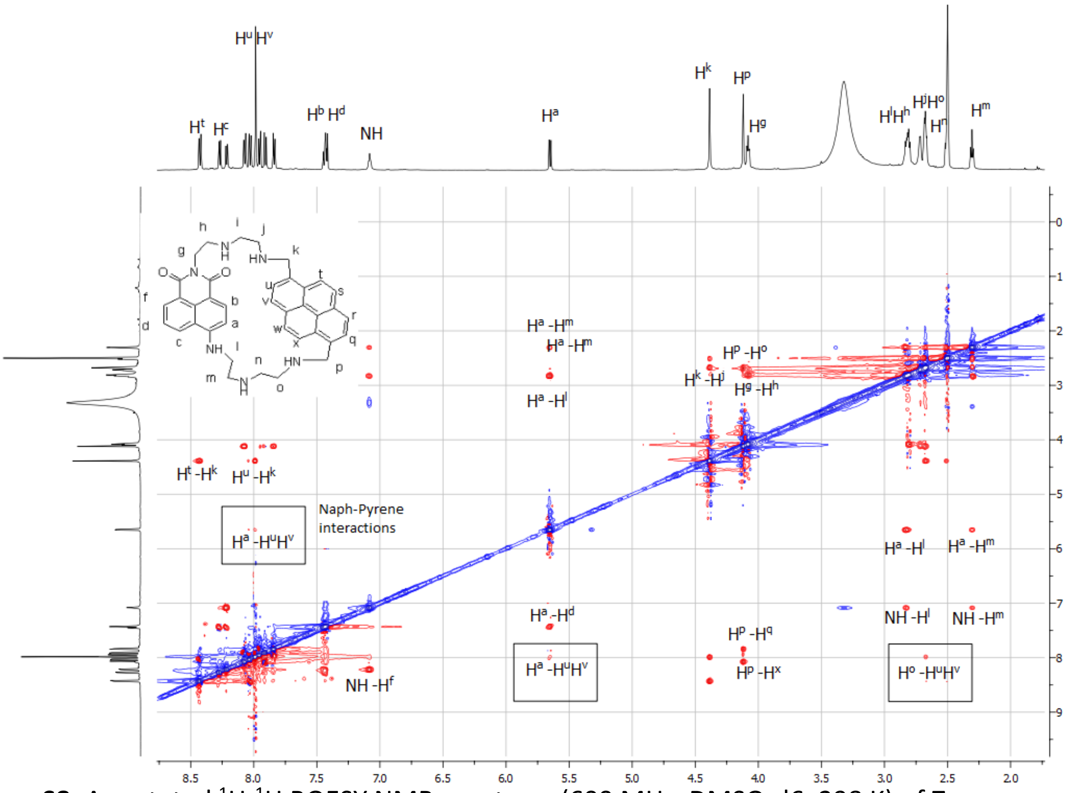

Figure S8. Annotated ^{1}H-^{1}H ROESY NMR spectrum (600 MHz, DMSO-d6, 298 K) of **7**.

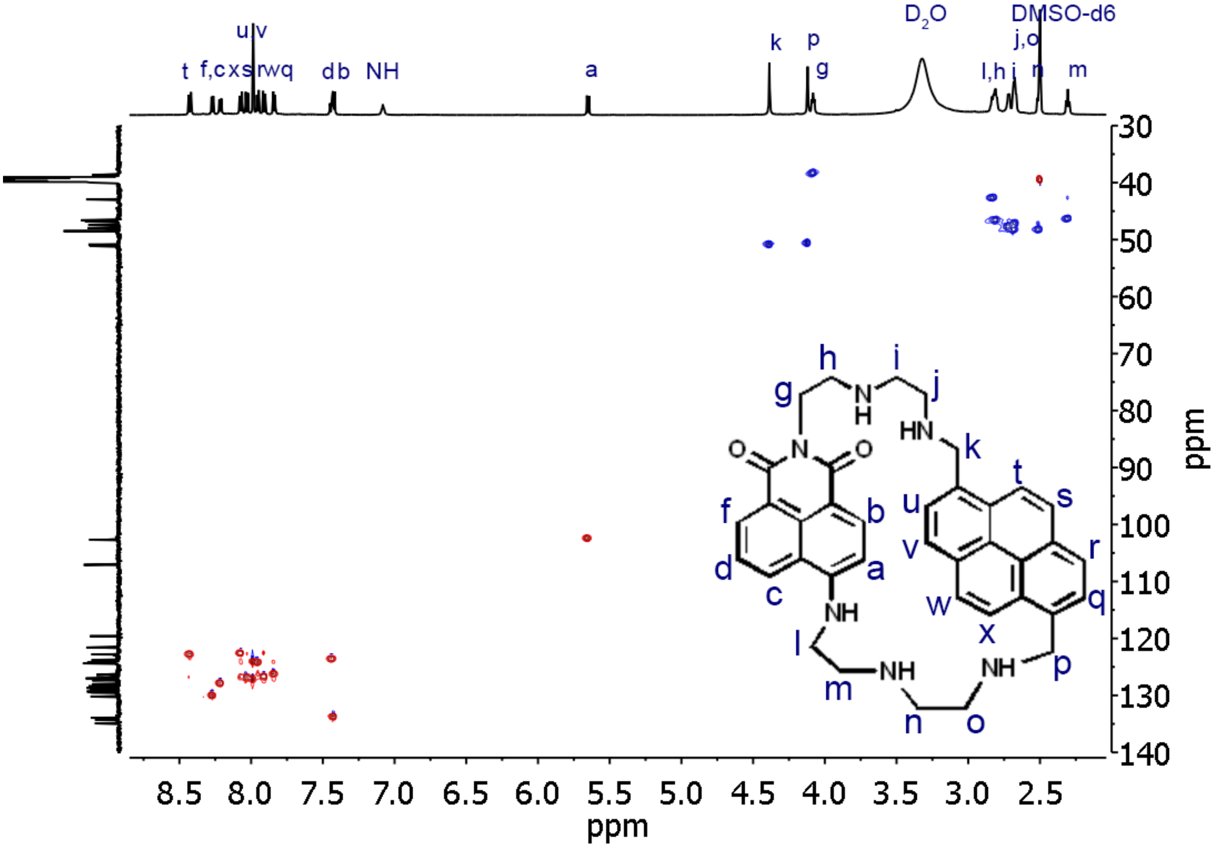

Figure S9. HSQC spectrum of compound **7** in DMSO-d_6

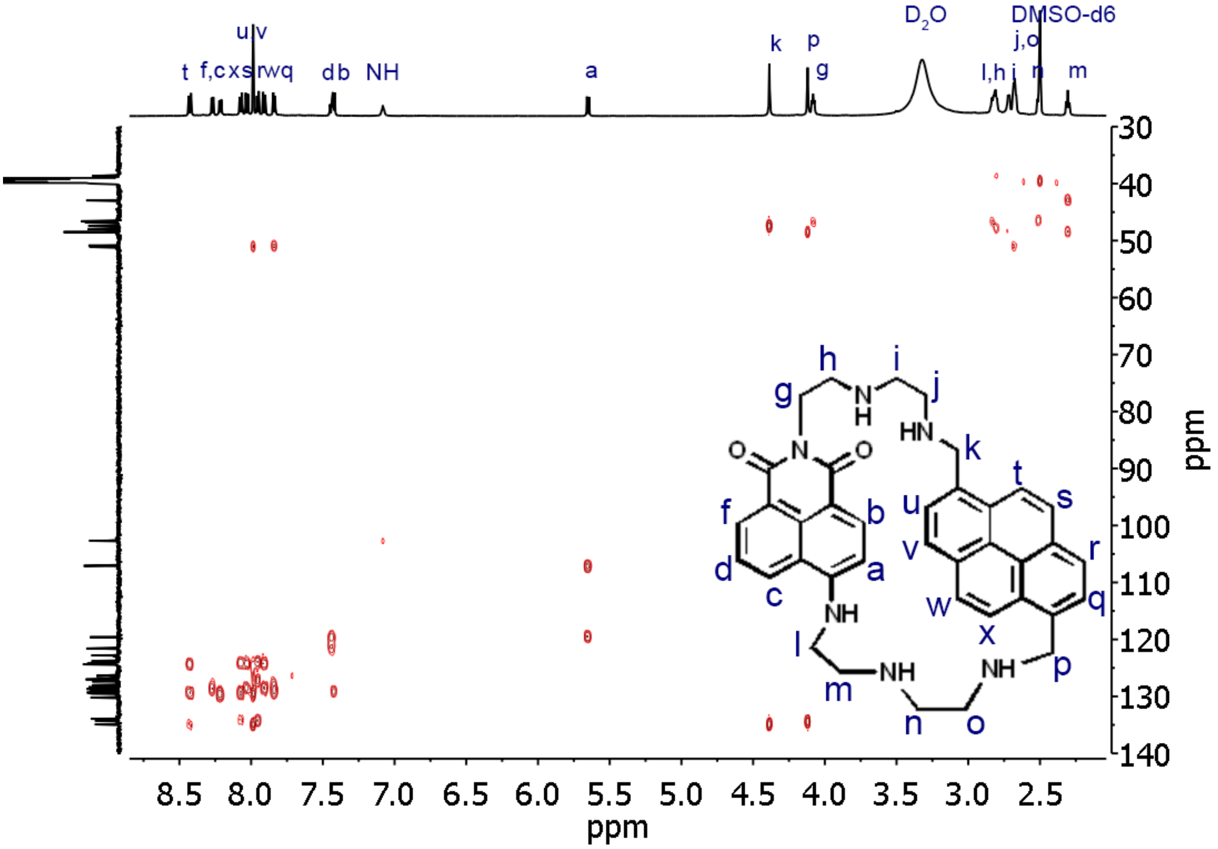

Figure S10. HMBC spectrum of compound **7** in DMSO-d_6

^{1}H NMR titrations

The titrations were carried out by sequential addition of sodium salts to the NMR tube containing the receptors followed by the measurements. The mixture of 25% DMSO in 10 mM TRIS buffer, 100 mM NaCl, pH 7.4 mixture was used because of solubility limitation of the receptors at 0.5 mM concentration. The following conditions were used: 0.5 mL of 0.5 mM solution of receptors in a 1:3 DMSO-d_6–D_2O (10 mM TRIS buffer, 100 mM NaCl, pH 7.4) mixture. AMP was dissolved in the same D_2O-based buffer (6.25 mM) were added as follows (equiv): 0.25; 0.5; 0.75; 1; 1.5; 2; 3, 5; 7; 10. The fitting was performed by HypNMR program.

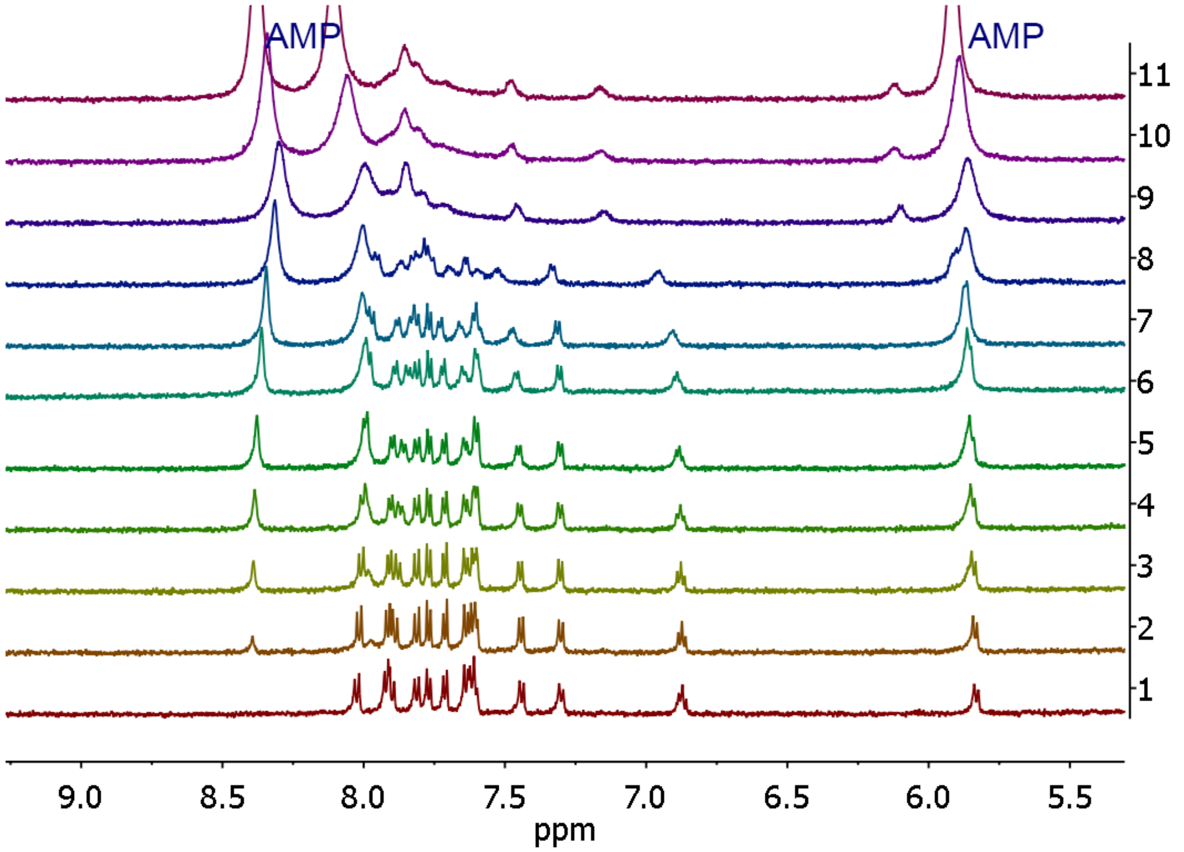

Figure S11. ^{1}H NMR titration of receptor **7** with AMP. The solution of **7** (0.5 mM) in a mixture of 25% DMSO in 10 mM TRIS buffer, 100 mM NaCl, pH 7.4 was added to NMR tube and ^{1}H spectrum was measured (spectrum 1). Spectra 2-11 correspond to the addition of different equiv of AMP (6.25mM) dissolved in the same buffer.

Fluorescence and UV-Vis titrations

Stock solutions of receptors with concentrations of 10^{-5} M in a 10 mM TRIS buffer containing 100 mM NaCl, pH 7.4 (2% DMSO) were prepared for binding studies. The titrant (nucleotide, 0.01M) was sequentially added to a 2 mL sample of the host stock solution in the spectrometric cell and the changes in the spectral features were monitored. The total number of data points was 20-40, depending on the strenght of complexation; for a presumed 1:1 and 1:2 complexation 30 points were usually measured. The following setup parameters were used for fluorescence titration experiments: ex. 350 nm, slit 2/2, em: 360-700 nm. The resulting data was imported in HypSpec program[1] and spectral changes were fitted to obtain stability constants with nucleotides.

	Fluorescence changes	Fitting graphic
ATP	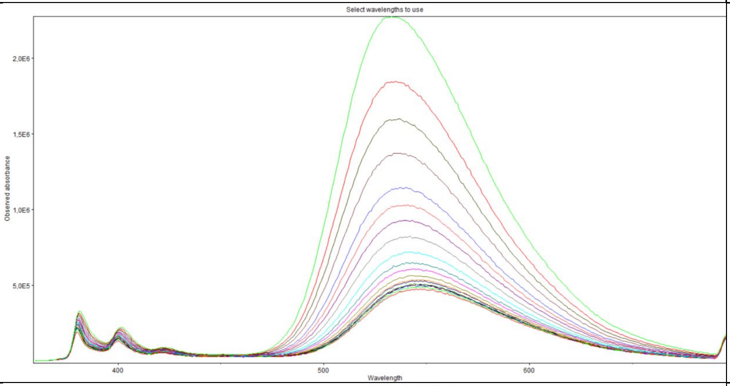	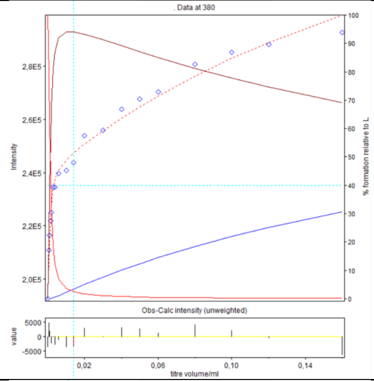
GTP	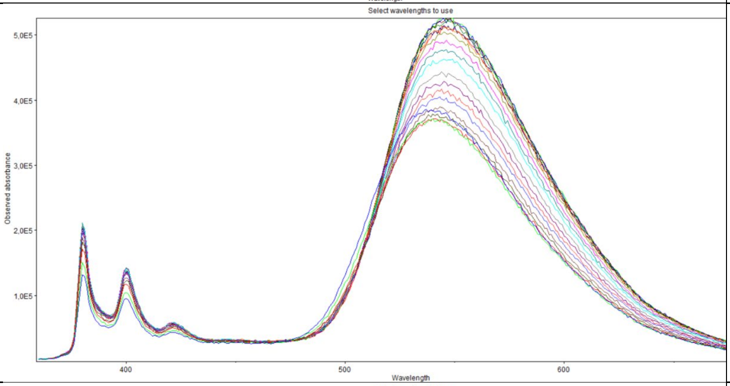	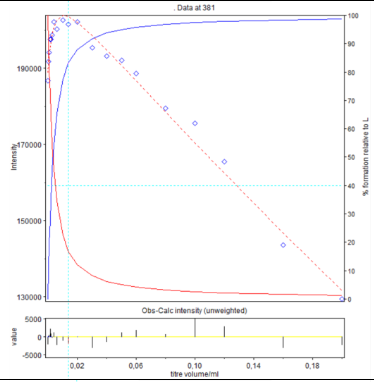
CTP	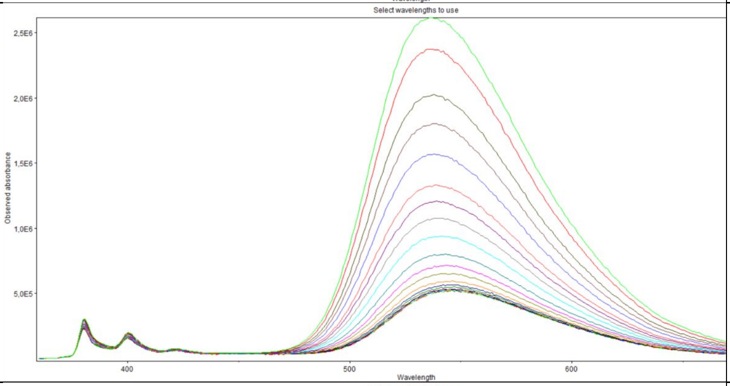	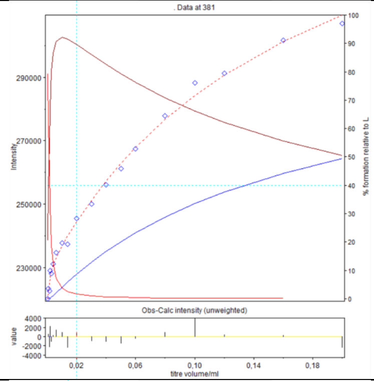
UTP	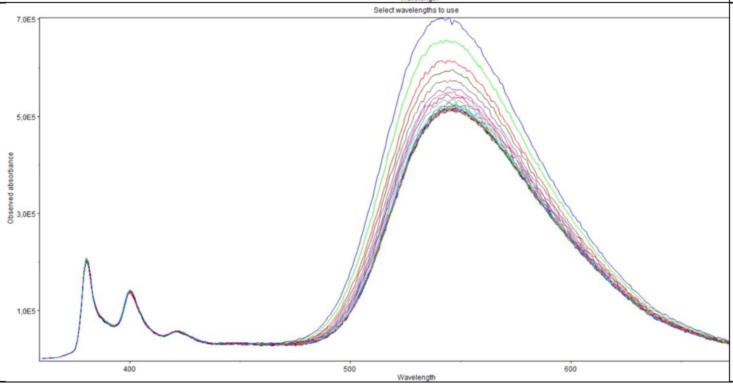	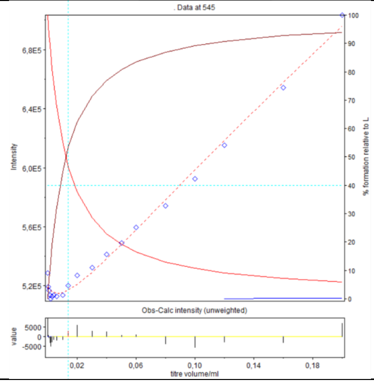

Figure 12. Fluorescence titration of **7** with nucleotides together with the fitting curves exported from HypSpec Program. Blue points represent the experimental data at selected wavelength, while the red-dotted line is the fitting curve.

The limit of detection (LOD)

The LOD (limit of detection) and LOQ (limit of quantitation) were calculated using the following formulas: LOD = 3.3 σ/S, LOQ = 10 σ/S. Where σ=the standard deviation of the analytical background response, and S = the slope of the calibration curve (plot of fluorescence intensity as a function of the nucleotide concentrations in the region of low analyte content). [2–4] To determine the standard deviation, the fluorescent intensity of 7 without any analyte was measured in 10 prepared probes (with concentration 1E-5M). Equation 1:

$$\sigma = \sqrt{\frac{1}{\mathrm{N}-1}\sum_{i=1}^{N}(m_i-\mu)^2}$$

where {m_1, m_2 , … , m_N} are the observed fluorescent intensities at certain wavelength values of the sample items, and μ is the mean value of these observations, while the denominator N stands for the number of probes. In our case, there were 10 samples, the standard deviation was 34866.20 with an average fluorescence intensity of 449606 a.u. at 530 nm.

There was a good linearity from 10^{-5} to milimolar concentration levels between fluorescent intensity data at 530 nm and concentrations of ATP. The linear equation (equation 2)

$$y = Sx + b$$

was found to be y= 2.44873E9x+ 2.44873E9 (Figure S13 a), where y is the fluorescent intensity data at 530 nm measured at given ATP concentration and x represents the concentration of ATP added. Thus, the detection limit and the limit of quantitation of **7** were calculated. LOD = 3.3 σ/S=3.3*34866.20/2.44873E9=4.6987E-05 (ca. 0.05 mM), LOQ = 3.3 σ/S=10*34866.20/2.44873E9= 1.42E-04 (ca. 0.15 mM).

The values LOD and LOQ for the ratiometric determination of ATP were calculated in a similar way. For this, the ratio of fluorescence intensities at 530 nm and 380 nm was used. In this case, the following values were calculated and obtained: σ= 0.188758, y= 7807.3x+ 1.8802 (Figure S13 b). Calculated values LOD= 7.98E-05 (ca. 0.08 mM) and LOQ= 2.42E-04 (ca. 0.25 mM) are somewhat different from those obtained above, which is associated with a larger error and lower sensitivity of the pyrene fragment.

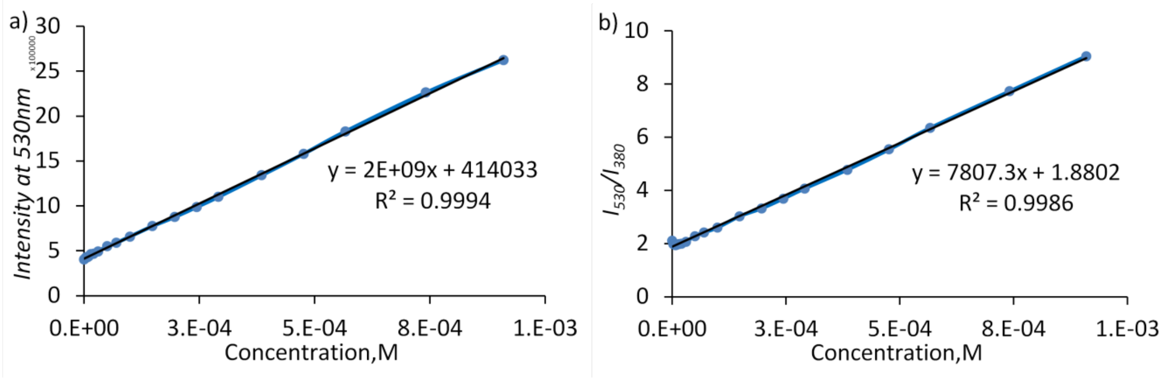

Figure S13. a) Fluorescence changes of receptor 7 (c=10^{-5} M) at 530 nm and b) changes in intensity ratio I_{530nm}/I_{380nm} with increasing amounts of ATP portion with corresponding trend lines.

UV-Vis titrations were carried out were carried out in the same manner as the fluorescence titrations. To a receptor solution with $2*10^{-5}$ M concentration, a solution of the receptor ($2\cdot10^{-5}$ M) and a nucleotide (0.02 M) was added in portions. At each step a spectrum was measured. All the spectra were combined by using HypSpec program and fitted to yield the binding constants.

	UV-Vis changes	Fitting graphic
ATP	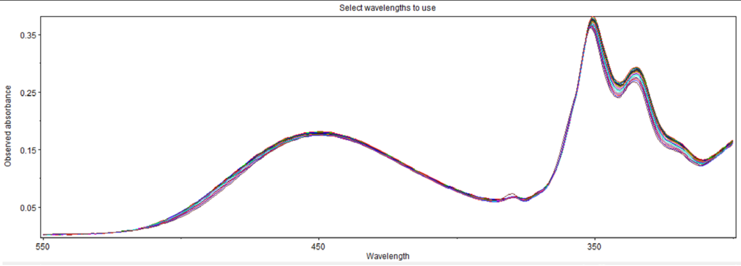	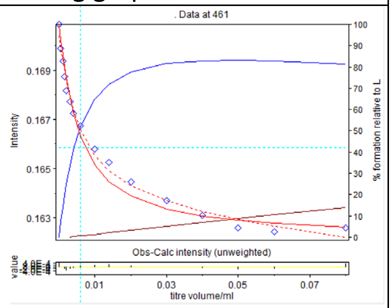
GTP	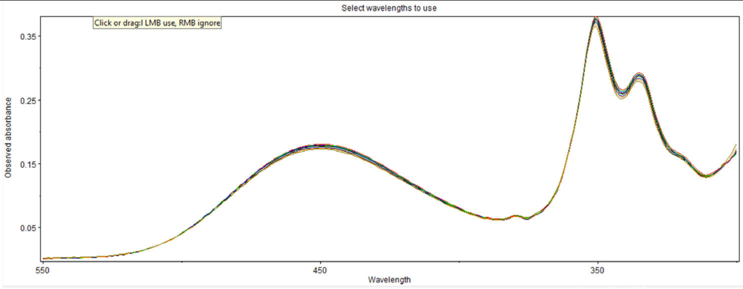	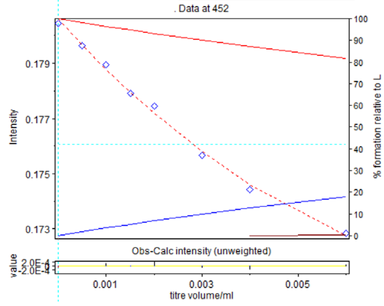
CTP	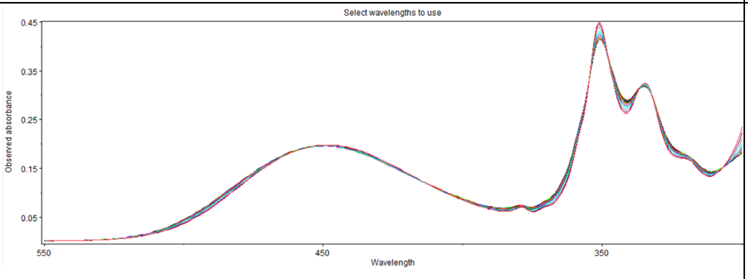	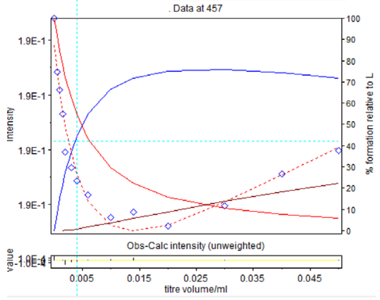
UTP	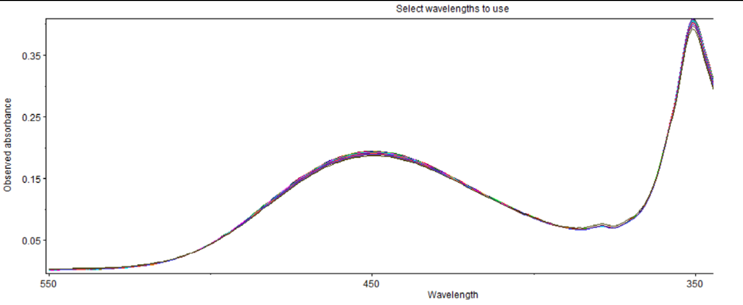	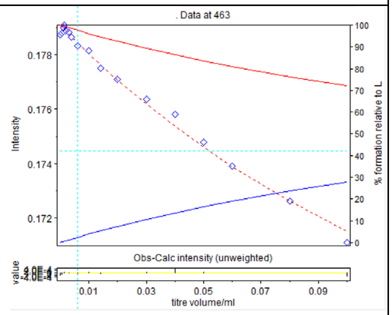

Figure 14. UV-Vis titration of **7** with nucleotides together with the fitting curves exported from HypSpec Program. Blue points represent the experimental data at selected wavelength, while the red-dotted line is the fitting curve.

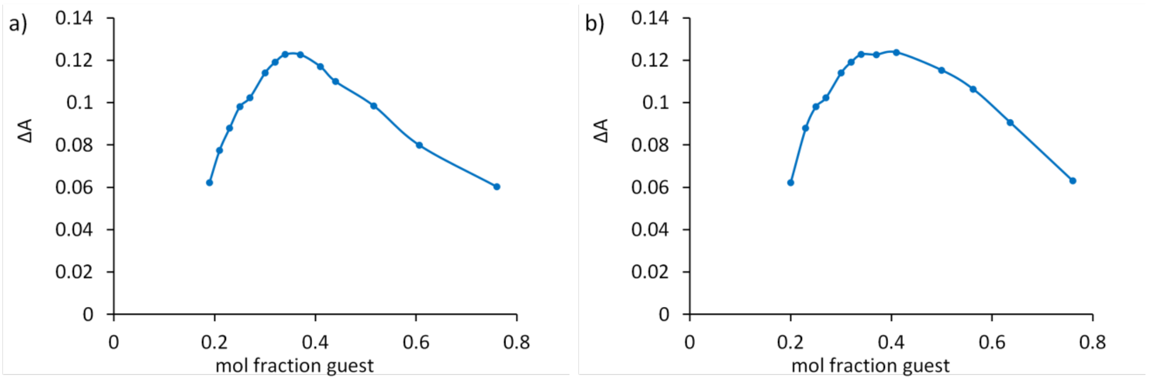

Figure S15. Job plot: stoichiometry determination of receptor **7** in the presence of a) ATP, b) CTP, indicating 1:2 major binding mode. Conditions: 0.02 mM receptor and nucleotide concentration in 10 mM TRIS buffer, 100 mM NaCl, pH 7.4 (2% DMSO).

Binding constants

Table S1. Binding constants of receptor **7** for nucleotides as determined from fluorescence and UV-Vis titrations in 10 mM TRIS buffer, 100 mM NaCl, pH 7.4 (2% DMSO) at 23°C.

**Nucleotides**	**Fluorescence**	**UV-Vis**
ATP	logK_{11} = 4.80(2)<br>logK_{12} = 2.39(3)	logK_{11} = 4.34(4)<br>logK_{12} = 2.36(4)
GTP	logK_{11} = 3.66(3)<br>logK_{12} < 2	logK_{11} = 3.54(2)<br>logK_{12} < 2
CTP	logK_{11} = 4.53(4)<br>logK_{12} = 2.55(3)	logK_{11} = 4.42(4)<br>logK_{12} = 2.82(4)
UTP	logK_{11} = 3.23(2)<br>logK_{12} < 2	logK_{11} = 2.61(3)<br>logK_{12} < 2

Competition experiment

The competition experiments were conducted as follows:

a) 10^{-5} M solution of the receptor in a buffered solution in 10 mM TRIS buffer, 100 mM NaCl, pH 7.4 (2% DMSO)was treated first with excess ATP (150 equiv) dissolved in the same buffer and fluorescence was measured before and after addition. The ratio of intensities (I/I_0) represents the relative fluorescence increase after addition of ATP (marked as "ATP" (red bars) below in Figure). Then the competing nucleotide (300 equiv) was added and the fluorescence of the resulting mixture (competing nucleotide with ATP (blue bars)) was measured.

b) 10^{-5} M solution of the receptor in a buffered solution in 10 mM TRIS buffer, 100 mM NaCl, pH 7.4 (2% DMSO)was treated first with excess of competing nucleotide (100 equiv) dissolved in the same buffer and fluorescence was measured before and after addition (coloured bars). Then ATP (50 equiv) was added and the fluorescence of the resulting mixture (competing nucleotide with ATP (red bars)) was measured.

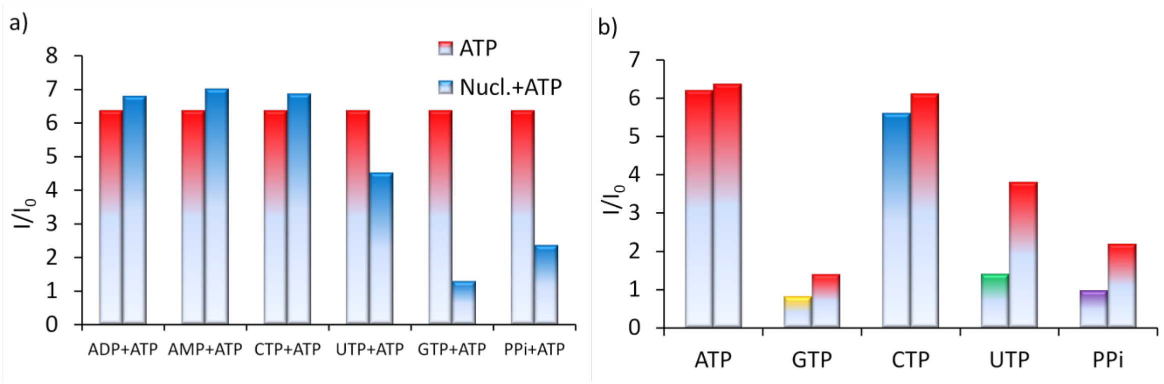

Figure S16. Normalized fluorescence intensity at 532 nm of receptor **7** (0.01 M) in the presence of a) 150 equiv of ATP(red bars) and after addition of competing nucleotide (300 equiv; blue bars); b) 100 equiv of a competing nucleotide (coloured bars) and after addition of ATP (50 equiv; red bars).

Quantum chemical calculations

Molecular modeling calculations were performed using program "PRIRODA" developed by D. Laikov.[1] For the calculations it was used a parametrized model "qn3" involving dispersion interactions. The host-guest model structures were generated by combining a preoptimized structure of the receptor with a nucleotide. Next, these starting geometries were obtained by a limited set of conformations from molecular dynamics. The criterion for convergence was a difference below 0.01 kcal/mol/Angstrom in the energy between two sequential structures. Searches for the relevant global minima were performed by calculating different nucleotide-to-receptor coordination modes.

Table S2. Cartesian coordinates for the optimized nucleoside-receptors complexes.

**7**H_2^{4+}*adenosine complex	**7**H_2^{4+}*cytidine complex
7 4.57982206 -0.84926260 -0.81123281	7 4.71369028 0.44730583 -0.56056565
6 -3.76442075 1.70413494 -2.63066578	6 -4.25686979 3.07714701 -2.16750979
1 -3.35079479 2.43678069 -1.94167519	1 -3.71036649 3.81223798 -1.58478475
6 -4.78688192 0.84854233 -1.90610600	6 -5.51853943 2.62190032 -1.43152893
1 -5.06633759 -0.00107196 -2.53299546	1 -6.17301083 2.07376218 -2.11418343
1 -5.69457293 1.40798521 -1.67368937	1 -6.08582258 3.44270229 -0.98996294
7 -2.72254634 0.85637045 -3.13677263	7 -3.45596266 1.88731992 -2.38661218
1 -3.02139974 0.22370961 -3.85856009	1 -3.79101872 1.39381790 -3.20811248
6 -1.38017666 1.17638457 -3.17798877	6 -2.03937054 1.91621363 -2.41186690
6 -0.83686435 2.22071648 -2.46331096	6 -1.39522004 1.35693121 -3.48433280
1 -1.47266376 2.92988658 -1.96025896	1 -1.96755874 1.00791216 -4.33603859
6 0.54298496 2.33308077 -2.31187296	6 0.00152447 1.23461926 -3.50651217
1 0.94896030 3.11125922 -1.68497491	1 0.49587262 0.79353982 -4.36571932
6 1.39130032 1.41930890 -2.87100363	6 0.74727792 1.67458332 -2.45289540
6 0.87848246 0.44029906 -3.74558353	6 0.11540427 2.25496101 -1.33452177
6 -0.50448179 0.31711879 -3.92697954	6 -1.27880490 2.38324785 -1.29619265
6 -0.97526824 -0.67244446 -4.82132244	6 -1.85581124 2.92396402 -0.12382508
1 -2.03348255 -0.77760112 -5.01025772	1 -2.91944218 3.04423618 -0.07300055
6 -0.12157492 -1.50449884 -5.48139858	6 -1.10472465 3.32231212 0.93657124
1 -0.50383556 -2.24874163 -6.17299795	1 -1.57121122 3.76516485 1.80964005
6 1.26049554 -1.39777434 -5.26152372	6 0.29270452 3.16868401 0.89487278
8 4.00198460 -1.15637147 -4.63822937	8 3.03622055 2.39614964 0.75018162
6 3.20276761 -0.41096932 -4.14072418	6 2.33889627 2.43680143 -0.23098139
6 1.74722803 -0.44832674 -4.40823984	6 0.87781399 2.63922477 -0.21252812

6	2.79864979	1.38305497	-2.46477103	6	2.21849489	1.55379605	-2.50045443
8	3.24940920	1.99506259	-1.52601123	8	2.82351661	0.89294654	-3.29716396
7	3.61764956	0.50476205	-3.16743708	7	2.89644098	2.25265670	-1.49419296
6	5.02358818	0.51260006	-2.83346581	6	4.30359459	2.56442881	-1.66385436
1	5.23209333	1.41756105	-2.27253866	1	4.58443451	3.22686934	-0.84851635
1	5.62286186	0.51273584	-3.74524307	1	4.45171022	3.09313607	-2.60832453
6	5.39570427	-0.70672894	-2.04060268	6	5.16147661	1.32822049	-1.66259086
1	5.21574068	-1.60106540	-2.63584256	1	5.03162956	0.76961905	-2.58631372
1	6.45042944	-0.68326604	-1.75627375	1	6.21912336	1.57266510	-1.54516280
6	4.94211864	-0.02524090	0.36198077	6	5.47671413	0.49428654	0.69711041
1	4.70276546	1.01791322	0.16434124	1	5.83156157	1.50883973	0.88456905
1	6.01773596	-0.12400194	0.51566601	1	6.34392071	-0.16210210	0.58052635
7	-4.22535324	0.31123456	-0.63799876	7	-5.12471581	1.63235939	-0.35427842
7	4.37046576	-2.02257490	1.60494232	7	3.75777149	-1.09754241	1.57155883
7	-5.74147081	-0.94302344	1.17608821	7	-6.21350670	0.45524815	2.52337694
6	4.16804218	-0.58644229	1.53935599	6	4.60880995	0.08234926	1.86973405
1	3.11143804	-0.40216017	1.38391697	1	3.93193722	0.89328724	2.12587571
1	4.46717215	-0.04209098	2.43954325	1	5.25128651	-0.14576682	2.72499847
6	3.46131253	-2.72974873	2.50816989	6	3.00856876	-1.62094235	2.77478099
1	3.85359430	-3.74766064	2.61306047	1	3.45076346	-2.58763742	3.02246928
1	3.46790957	-2.29171872	3.50750279	1	3.20281243	-0.94506437	3.60144830
6	2.04769206	-2.81499386	1.98734891	6	1.54167783	-1.76127982	2.50830698
6	1.79736602	-3.70523810	0.95129311	6	1.07657290	-2.96029329	1.98486185
6	0.51949632	-3.92469716	0.48418072	6	-0.27490601	-3.19739676	1.83988869
6	-0.55637825	-3.23481965	1.03313637	6	-1.19837105	-2.24616313	2.25897431
6	-0.32620880	-2.29031348	2.04546452	6	-0.74974865	-1.00866497	2.75260544
6	0.98444653	-2.08879209	2.53531122	6	0.63298440	-0.74992651	2.84509778
6	1.15872157	-1.15560460	3.62317324	6	1.05110526	0.54443491	3.32058334
1	2.14627194	-1.00783324	4.03606367	1	2.10317802	0.78051358	3.36835361
6	0.13185728	-0.45952234	4.12686205	6	0.16486210	1.46969485	3.70785904
1	0.29017940	0.24598959	4.93594646	1	0.50094479	2.43253040	4.07715178
6	-1.20713770	-0.63225156	3.62395787	6	-1.24935138	1.20541561	3.68110251
6	-2.28234959	0.06774059	4.16067839	6	-2.16824770	2.14441323	4.14682531
1	-2.10823655	0.79745638	4.94413948	1	-1.81632578	3.09737897	4.52761173
6	-3.56958818	-0.20237659	3.73864985	6	-3.51368356	1.85095572	4.16012859
1	-4.39742327	0.31824175	4.20851421	1	-4.20655251	2.58236837	4.56506443
6	-3.82329893	-1.15623796	2.76139307	6	-3.98609614	0.62394905	3.69600296
6	-2.74853969	-1.82123327	2.15889883	6	-3.08453870	-0.33019078	3.19937396
6	-1.43208456	-1.56949198	2.60001135	6	-1.70259929	-0.03556790	3.19631314
6	-1.90835583	-3.47897291	0.60266912	6	-2.61373758	-2.51534986	2.24357462
1	-2.07042217	-4.24062204	-0.15148388	1	-2.94882441	-3.48190331	1.87720335
6	-2.94248438	-2.80693817	1.12523127	6	-3.50300956	-1.61967230	2.69740319
1	-3.95029974	-3.01903486	0.79607069	1	-4.54977036	-1.89123833	2.72061181
6	-5.25414181	-1.49988532	2.44266343	6	-5.43825436	0.33853143	3.83810544
1	-5.87516451	-1.15985072	3.27711391	1	-5.91945982	1.04107130	4.51823616
1	-5.37542582	-2.58524799	2.38216376	1	-5.64651680	-0.67947251	4.17667532
6	-5.86605692	0.50428903	1.14218235	6	-6.26150846	1.82258832	1.89753878
1	-6.69985056	0.77521932	0.48576424	1	-7.18146372	1.87657392	1.30470693
1	-6.06448650	0.96447647	2.11576629	1	-6.34216166	2.54301214	2.71497011
6	-4.57836151	1.08507049	0.58208084	6	-5.04850674	2.16070390	1.05154872
1	-3.76520467	0.94132054	1.28782868	1	-4.14765835	1.75983739	1.51076829
1	-4.65717554	2.14662910	0.34261689	1	-4.95638657	3.24618721	0.99533898

1 -6.63830566 -1.37540746 0.96117473	1 -7.18472290 0.16489048 2.71890187
1 -3.18349147 0.22479174 -0.68672597	1 -4.19919157 1.27336299 -0.66498709
1 5.32557487 -2.21645188 1.90335023	1 4.32594967 -1.84733665 1.16705060
1 -4.28273630 2.26095200 -3.42487288	1 -4.57472610 3.57765269 -3.09094310
1 3.58211637 -0.67706299 -1.00379980	1 3.74307799 0.68279338 -0.37527695
6 1.78126371 1.80204511 2.21013498	6 3.34665537 -2.03948426 -2.13844204
6 -0.09150855 4.92660332 1.71865261	6 2.84059596 -5.09046984 -4.16227102
6 0.96971393 3.87173009 1.63379538	6 3.03584647 -4.20249128 -2.97002459
6 1.41580307 1.83475518 0.74566841	6 2.13611865 -2.72983551 -1.55152321
6 0.94514787 2.91079330 2.78405857	6 3.70792961 -2.90701151 -3.32113385
6 -0.60233891 -0.69359189 -0.83453536	6 -1.40691590 -1.76234269 -1.58615518
6 -0.88156724 0.95575356 0.46432200	6 -0.23396160 -2.33406520 -1.93167996
6 0.66151440 -0.25228113 -0.48383978	6 -1.33301163 -0.74230474 -0.59562838
6 -0.69591486 -1.76019001 -1.74193859	6 0.99016368 -0.78747666 -0.58991975
6 1.60177565 -1.82408154 -1.74549413	1 2.41871262 -6.05401278 -3.85352683
1 0.00794968 5.62551165 0.88015473	1 5.12346888 -2.54440022 -1.66425765
1 3.23953128 2.61496353 3.14600849	1 2.13189721 -4.62980175 -4.86073732
1 -1.08429718 4.46650982 1.63851869	1 -2.27209711 0.14484455 0.89208019
1 -1.84073484 -3.09615088 -2.69591713	1 3.12487841 -1.01778281 -2.42498779
1 1.55316925 0.83413339 2.64846396	1 3.66322446 -4.72654724 -2.24756169
1 1.94408619 4.36075640 1.62055886	1 2.38919330 -3.04532456 -0.53451073
1 2.29825521 1.68504333 0.13361636	1 3.27720571 -2.49383307 -4.23729467
1 -0.07893489 2.56228161 2.94878340	1 5.29496908 -3.74004984 -4.01693296
1 1.10945594 4.27541924 4.11398077	1 -3.20611882 -0.95458680 0.02774093
1 -1.29762304 1.75233626 1.06207180	8 2.08675575 -0.33542582 -0.24980637
1 -2.67027664 -2.11560512 -1.57245994	7 -2.48251081 -0.24904977 -0.01313382
1 2.48647857 -2.33353400 -2.12736797	7 0.94339103 -1.89417076 -1.42121160
7 0.46733424 0.81791973 0.34531477	7 -0.18183579 -0.26923001 -0.18166433
7 -1.56108165 0.08647016 -0.21488100	8 4.39438820 -2.07457900 -1.21351540
7 -1.89031947 -2.21967959 -2.19825864	8 5.07735205 -3.03241110 -3.38789105
7 1.79367673 -0.80220515 -0.91919541	8 1.84631956 -3.79184866 -2.35060716
7 0.44093004 -2.29407668 -2.18076468	8 4.11998892 -5.21589899 -4.72833586
8 3.12437654 2.10840058 2.33117270	1 4.13438225 -5.88406754 -5.42501497
8 1.50734949 3.41114569 3.93633866	1 -0.61978209 -4.14799595 1.44636047
8 0.82447445 3.05095196 0.51548731	1 4.69566059 -0.53367567 -0.89096147
8 0.07163298 5.56127834 2.96365428	1 -5.77486706 0.84344202 -0.39590922
1 -0.44809902 6.37164021 3.00529408	1 1.77926254 -3.74442959 1.72183549
1 0.34719113 -4.64456081 -0.30687335	1 0.91376519 3.47350597 1.72727382
1 4.63342810 -1.81570756 -0.46782637	1 -0.16888992 -3.17809582 -2.60539246
1 -4.60356140 -0.62247682 -0.42756638	1 -2.34833264 -2.11619639 -1.98074162
1 2.62279177 -4.26591015 0.52759492	1 3.05865598 -0.82615465 0.84965932
1 1.95782673 -2.06341934 -5.75757647	1 -5.83251429 -0.23621282 1.87212932
1 -5.01764727 -1.17341018 0.48045802	
1 4.16300678 -2.38053536 0.66168982	Energy = -2835.2938304712
Energy = -2907.4588179044	

References

1. Laikov, D. N.; Ustynyuk, Y. A., PRIRODA-04: a quantum-chemical program suite. New possibilities in the study of molecular systems with the application of parallel computing. *Russ Chem Bull* **2005,** 54, (3), 820-826.
